# In-hospital development of new-onset frailty and discharge outcomes among patients with sepsis: a Korean multicenter registry study

**DOI:** 10.1186/s40560-026-00907-1

**Published:** 2026-07-13

**Authors:** Eunjeong Choi, Kipoong Kim, Woo Hyun Cho, Jin Ho Jang, Ryoung-Eun Ko, Gee Young Suh, Chae Man Lim, Hye Ju Yeo

**Affiliations:** 1 Transplant Research Center, Research Institute for Convergence of Biomedical Science and Technology, Pusan National University Yangsan Hospital, Yangsan, Republic of Korea; 2 Department of Statistics, Changwon National University, Changwon, Korea; 3 Department of Internal Medicine, School of Medicine, Pusan National University, Geumo-Ro 20, Beomeo-Ri, Mulgeum-Eup, Yangsan-Si, Gyeongsangnam-Do 626-770, Busan, Republic of Korea; 4 Department of Critical Care Medicine, Samsung Medical Center, Sungkyunkwan University School of Medicine, Seoul, Republic of Korea; 5 Department of Critical Care Medicine, Asan Medical Center, Seoul, Republic of Korea

**Keywords:** Sepsis, Frailty, Functional outcomes, Critical illness, Discharge destination

## Abstract

**Background:**

Frailty is an important marker of vulnerability and poor outcomes in patients with sepsis. Although prior studies have primarily focused on pre-existing frailty or post-discharge outcomes, little is known about the in-hospital development of frailty among patients who are non-frail at baseline.

**Methods:**

We conducted a multicenter cohort study using the Korean Sepsis Alliance registry, a prospectively maintained database of adult patients with sepsis across 15 hospitals in South Korea. Analyses were restricted to patients without baseline frailty, defined as a premorbid Clinical Frailty Scale score < 5. The primary analysis categorized patients as remaining non-frail at discharge, having new-onset frailty at discharge, or dying during hospitalization. Multinomial logistic regression with prespecified covariates was used to evaluate clinical factors associated with these mutually exclusive outcomes, with in-hospital death treated as a competing outcome. Among hospital survivors, discharge destination was described according to discharge frailty status. In an exploratory analysis restricted to patients with new-onset frailty, we evaluated clinical factors associated with home discharge.

**Results:**

Among 6336 patients without baseline frailty, 1453 (22.9%) had new-onset frailty and 1527 (24.1%) died during hospitalization. Older age (per 1-SD increase, adjusted odds ratio [aOR], 1.38), higher Charlson Comorbidity Index (per 1-SD increase, aOR, 1.27), higher Sequential Organ Failure Assessment score (per 1-SD increase, aOR, 1.41), intensive care unit admission (aOR, 1.47), and pulmonary infection (aOR, 1.77) were associated after adjustment with new-onset frailty (all *p* < 0.001). Several of these factors were also associated with in-hospital death, with a larger adjusted estimate for SOFA score. Among hospital survivors with new-onset frailty, 42.8% were discharged home. In exploratory analyses restricted to patients with new-onset frailty, higher Sequential Organ Failure Assessment score, solid malignancy, and nursing home- or nursing hospital-acquired infection were associated with a lower likelihood of home discharge. In sensitivity analyses using inverse probability of censoring weighting, the overall pattern of associations for new-onset frailty remained generally consistent.

**Conclusions:**

New-onset frailty and in-hospital death were common among patients with sepsis who were non-frail at baseline. Assessment of frailty at admission and discharge may help characterize functional decline and inform discharge planning after sepsis.

**Supplementary Information:**

The online version contains supplementary material available at 10.1186/s40560-026-00907-1.

## Background

Sepsis remains a leading cause of morbidity and mortality worldwide and is increasingly recognized as a condition associated with long-term functional impairment among survivors [1–5]. Although advances in acute management have improved short-term survival, many patients experience persistent physical and functional decline after hospitalization, highlighting the growing importance of functional outcomes and recovery in sepsis survivors.

Frailty, a state of decreased physiologic reserve and increased vulnerability to stressors, has emerged as an important marker of poor outcomes in critically ill patients [6–8]. In patients with sepsis, frailty has been consistently associated with increased mortality, prolonged hospitalization, and poor functional recovery. Frailty is increasingly recognized as a dynamic condition rather than a fixed baseline characteristic. In community-dwelling older adults, Gill et al. demonstrated frequent transitions between frailty states over time, establishing frailty as a potentially modifiable and evolving condition [9]. In critically ill patients, prior studies have described functional trajectories before and after intensive care unit (ICU) admission, newly acquired or worsening frailty after critical illness, and frailty-related care processes during recovery using instruments such as the Clinical Frailty Scale (CFS) and Frailty Index [10–13].

In sepsis, however, most studies have focused on pre-existing frailty at admission or long-term functional outcomes after discharge [4, 14]. In contrast, the in-hospital development of frailty among patients who were non-frail before sepsis remains insufficiently characterized. Evaluating new-onset frailty at discharge may provide insight into functional deterioration occurring during the acute hospitalization phase, before post-discharge recovery or rehabilitation can be assessed.

Evaluating new-onset frailty during hospitalization is methodologically challenging because discharge frailty can be assessed only among hospital survivors. In-hospital death precludes assessment of discharge frailty and represents a competing outcome. Therefore, analyses that exclude patients who die before discharge may provide an incomplete or biased picture of frailty development after sepsis [15–17].

In this study, we used a multicenter sepsis registry to evaluate new-onset frailty among patients hospitalized with sepsis who were non-frail at baseline. Specifically, we aimed to (1) quantify the frequency of new-onset frailty at discharge, (2) evaluate prespecified clinical factors associated with new-onset frailty while accounting for in-hospital death as a competing outcome, and (3) describe discharge destination among hospital survivors according to discharge frailty status and explore clinical factors associated with home discharge among patients with new-onset frailty.

## Methods

### Study design and data source

We conducted a multicenter cohort study using the Korean Sepsis Alliance (KSA) registry, a prospectively maintained database of adult patients hospitalized with sepsis or septic shock across 15 tertiary and university-affiliated hospitals in South Korea. For this study, we performed a retrospective analysis of prospectively collected data from September 2019 to December 2023. The registry includes standardized demographic, clinical, laboratory, and outcome data collected at predefined time points. This study was approved by the institutional review boards of participating centers, and the requirement for informed consent was waived because de-identified registry data were used (Pusan National University Yangsan Hospital, IRB No. 05-2019-092). Details of participating centers and institutional review board approvals are provided in Supplementary Table 1.

### Study population

We included adult patients aged ≥ 19 years who were hospitalized with sepsis during the study period. For the primary analysis, we restricted the cohort to patients without baseline frailty, defined as a CFS score < 5 [6, 8, 18], reflecting premorbid functional status before the onset of sepsis. Patients without baseline frailty were categorized into three mutually exclusive in-hospital outcomes:

- (1) remaining non-frail at discharge (CFS < 5),
- (2) new-onset frailty at discharge (CFS ≥ 5), or
- (3) in-hospital death.

Because discharge frailty status is observable only among hospital survivors, in-hospital death was included as a mutually exclusive competing outcome rather than excluding these patients from the primary analysis.

### Frailty assessment

Frailty was assessed using the 9-point CFS. In the KSA registry, both baseline and discharge CFS were recorded according to standardized registry instructions. Baseline CFS was assessed at admission by physicians or trained research nurses and was intended to reflect the patient’s premorbid functional status before the onset of sepsis, rather than functional status after sepsis-related deterioration. Baseline CFS was determined primarily through direct patient interview when possible. If the patient was unable to provide reliable information, caregiver interview was performed. When neither patient nor caregiver information was sufficient, the assessors reviewed the medical records and selected the CFS category that best corresponded to the patient’s functional status before clinical deterioration due to sepsis.

Discharge CFS was assessed on the day of hospital discharge among hospital survivors by physicians or trained research nurses using the same registry protocol. Discharge CFS reflected the patient’s functional status at the end of hospitalization. Assessors reviewed the medical records and clinical information and selected the CFS category that best corresponded to the patient’s estimated functional status at discharge. The KSA registry provided standardized instructions for coding CFS from 1 to 9 for both baseline and discharge assessments. Baseline and discharge CFS were assessed by physicians or research nurses trained in registry data collection. The central coordinating institution provided training on registry data collection and data entry, including standardized instructions for CFS coding. However, formal CFS-specific certification, internal validation of CFS scoring, and inter-rater reliability testing across participating centers were not performed. Frailty was defined as CFS ≥ 5, a commonly used threshold in studies of critically ill patients [6, 8, 18].

### Outcomes

The primary outcome was a three-category in-hospital outcome among patients without baseline frailty: remaining non-frail at discharge, new-onset frailty at discharge, or in-hospital death. Discharge destination was evaluated among hospital survivors and categorized as home versus non-home discharge. Discharge destination was first described according to discharge frailty status. To explore heterogeneity in discharge outcomes among patients with new-onset frailty, home discharge was additionally evaluated in an exploratory analysis restricted to hospital survivors with new-onset frailty at discharge.

### Statistical analysis

Categorical variables are presented as numbers and percentages, and continuous variables as means with standard deviations or medians with interquartile ranges, as appropriate. Baseline characteristics were compared across the three in-hospital outcome groups. Comparisons across groups were performed using the chi-square test or Fisher’s exact test for categorical variables and analysis of variance or the Kruskal–Wallis test for continuous variables, as appropriate.

Multivariable models included prespecified covariates selected based on clinical relevance, prior literature, and availability in the registry: age, sex, body mass index (BMI), Charlson Comorbidity Index (CCI), Sequential Organ Failure Assessment (SOFA) score at admission, septic shock, ICU admission, infection acquisition setting, and site of infection. To account for the competing nature of in-hospital death, multinomial logistic regression was used with remaining non-frail as the reference outcome. This multinomial framework allowed simultaneous evaluation of new-onset frailty and in-hospital death within the same analytic cohort. Adjusted odds ratios (ORs) and 95% confidence intervals (CIs) were estimated for both outcomes using prespecified clinical covariates. Because analyses were restricted to patients without baseline frailty (CFS < 5), baseline CFS was not included as a covariate in the regression models. Baseline CFS values were presented descriptively to characterize premorbid functional vulnerability within the non-frail range.

Discharge destination was summarized descriptively according to discharge frailty status among hospital survivors. To explore heterogeneity in discharge outcomes among patients with new-onset frailty, an exploratory multivariable logistic regression analysis was performed among hospital survivors with new-onset frailty at discharge. Home discharge was the dependent variable, and prespecified baseline and early clinical characteristics were included as covariates. This analysis was interpreted as exploratory and survivor-conditional because it was restricted to hospital survivors with observed discharge frailty status and discharge destination.

As a sensitivity analysis, inverse probability of censoring weighting (IPCW) was applied among hospital survivors with observed discharge frailty status. The probability of survival to discharge was estimated using baseline and early clinical characteristics, and inverse probability weights were used to assess whether the associations between clinical factors and new-onset frailty were consistent after accounting for potential selection by survival. Patients with missing values for variables included in each model were excluded from the corresponding analysis. Statistical significance was defined as a two-sided *p* value < 0.05. All analyses were conducted using R software version 4.5.2. To further address the potential influence of acute-phase severity on frailty transition, we performed exploratory subgroup analyses stratified by ICU admission status and septic shock status. Within each stratum, multinomial logistic regression was repeated using the same three-category outcome definition and the same prespecified covariates as in the primary analysis, except that the corresponding stratifying variable was omitted from the model. These analyses were intended to assess the qualitative consistency of associations across clinically relevant severity subgroups.

## Results

### Study population and in-hospital outcomes

**Fig. 1:**
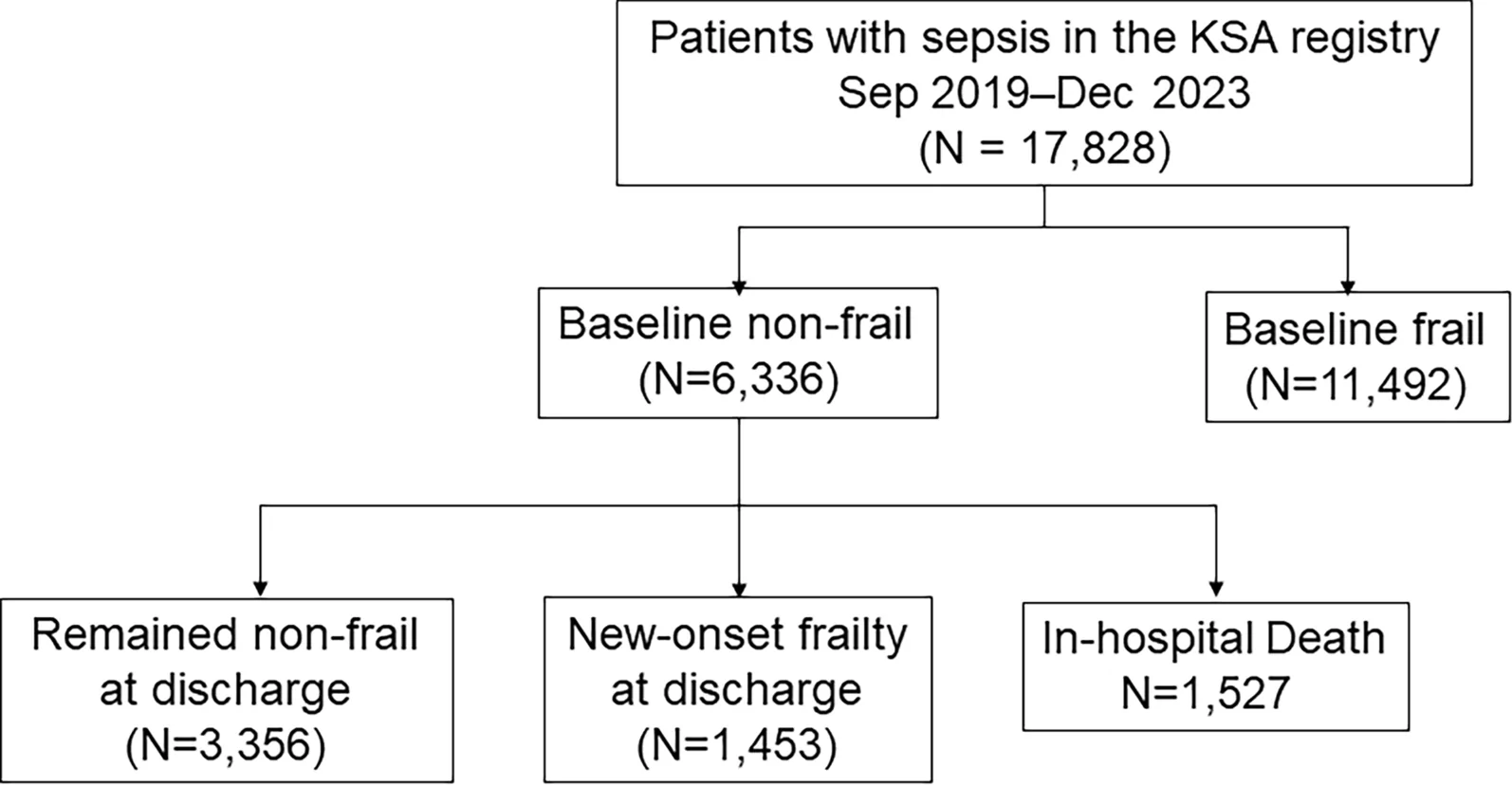
Study population and in-hospital outcomes. Flow diagram of the study population. Patients were stratified according to baseline frailty status. Among those without baseline frailty (Clinical Frailty Scale [CFS] < 5), in-hospital outcomes were categorized as remaining non-frail at discharge, new-onset frailty at discharge (CFS ≥ 5), or in-hospital death

**Fig. 2:**
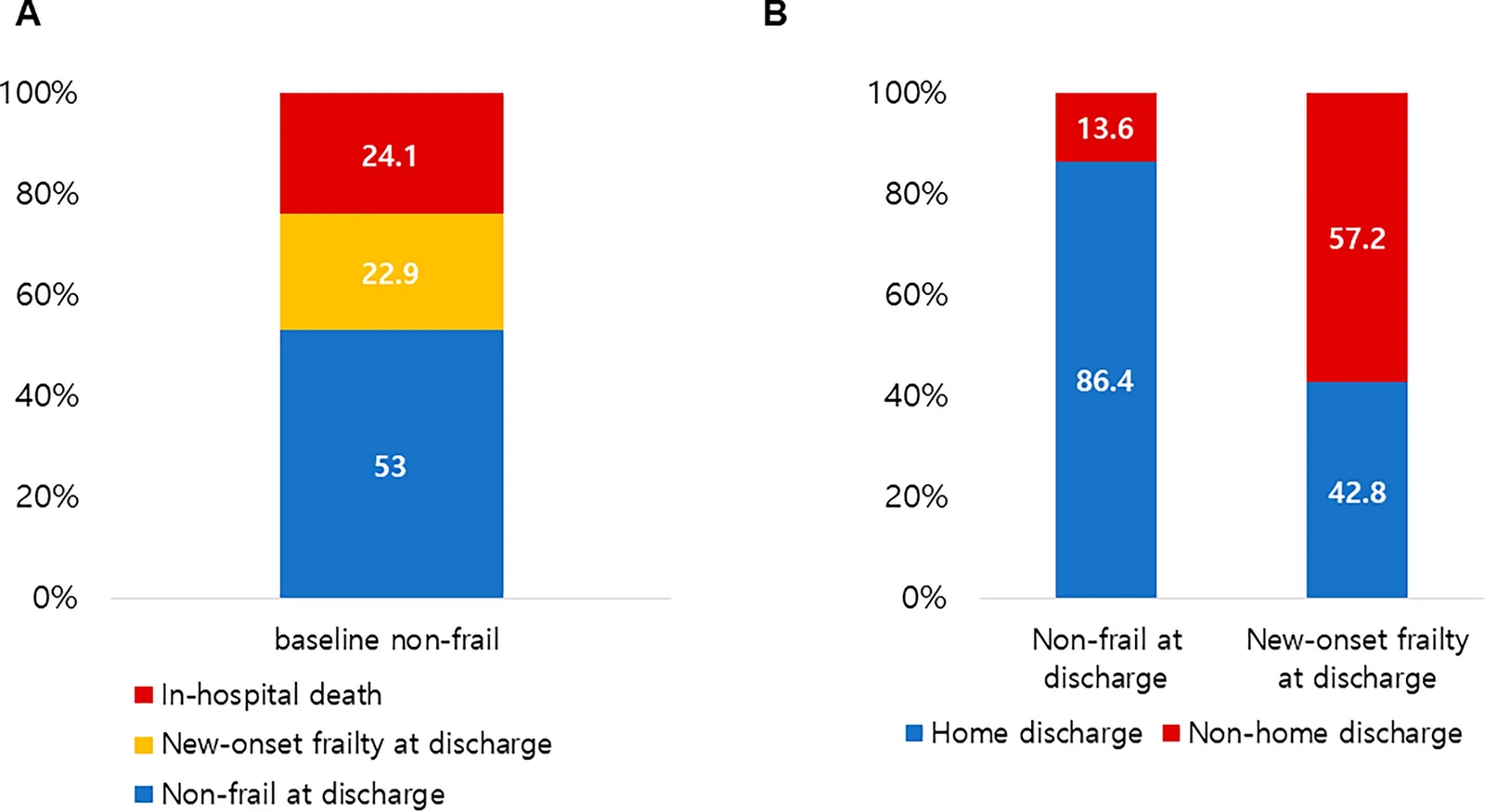
In-hospital outcomes and discharge destination among patients without baseline frailty. **A** Three-category in-hospital outcomes among patients without baseline frailty. **B** Discharge destination among hospital survivors according to discharge frailty status. Panel A includes all patients in the primary analytic cohort and shows mutually exclusive outcomes: remaining non-frail at discharge, new-onset frailty at discharge, or in-hospital death. Panel B is restricted to hospital survivors and shows discharge destination, categorized as home versus non-home discharge

Among 17,828 patients enrolled in the Korean Sepsis Alliance registry, 6,336 (35.5%) were non-frail at baseline and comprised the primary analytic cohort, while 11,492 (64.5%) were frail at baseline (Fig. 1). Among patients without baseline frailty, 3,356 (53.0%) remained non-frail at discharge, 1,453 (22.9%) had new-onset frailty at discharge, and 1,527 (24.1%) died during hospitalization (Fig. 2A). Among hospital survivors, home discharge was more frequent among patients who remained non-frail than among those with new-onset frailty at discharge (86.4% vs 42.8%), whereas non-home discharge was more frequent among patients with new-onset frailty (57.2% vs 13.6%; Fig. 2B).

### Baseline characteristics

Baseline characteristics stratified by the three in-hospital outcome groups are shown in Table 1. Compared with patients who remained non-frail, patients with new-onset frailty and those who died during hospitalization were older (64.9 ± 14.1, 70.3 ± 12.8, and 68.4 ± 13.0 years, respectively; *p* < 0.001) and had higher Charlson Comorbidity Index scores (4.7 ± 2.6, 5.5 ± 2.5, and 5.5 ± 2.7; *p* < 0.001). Baseline CFS values also differed across groups within the non-frail range (2.8 ± 1.1, 3.1 ± 1.0, and 3.2 ± 1.0; *p* < 0.001). SOFA scores and the frequency of septic shock were higher among patients with new-onset frailty and those who died during hospitalization. SOFA scores were 5.4 ± 2.6 in the remained non-frail group, 6.4 ± 2.9 in the new-onset frailty group, and 7.9 ± 3.4 in the in-hospital death group (*p* < 0.001). Septic shock was also more frequent in these groups (16.4%, 21.0%, and 29.4%, respectively; *p* < 0.001). ICU admission occurred in 34.3% of patients who remained non-frail, 43.6% of those with new-onset frailty, and 54.7% of those who died during hospitalization (*p* < 0.001).

**Table 1:** Baseline characteristics of patients without baseline frailty according to in-hospital outcomes

Variables	Remained non-frail (*n* = 3356)	New-onset frailty (*n* = 1453)	In-hospital death (*n* = 1527)	*p*
Demographics				
Age	64.9 ± 14.1	70.3 ± 12.8	68.4 ± 13.0	< 0.001
Male sex	1932 (57.6)	870 (59.9)	1013 (66.3)	< 0.001
BMI	22.4[20.0–25.1]	22.4[19.7–24.9]	22.0[19.5–25.1]	0.127
Baseline health status				
CCI	4.7 ± 2.6	5.5 ± 2.5	5.5 ± 2.7	< 0.001
CFS	2.8 ± 1.1	3.1 ± 1.0	3.2 ± 1.0	< 0.001
Severity				
SOFA	5.4 ± 2.6	6.4 ± 2.9	7.9 ± 3.4	< 0.001
Septic shock	550 (16.4)	305 (21.0)	449 (29.4)	< 0.001
Comorbidity				
Diabetes mellitus	1033 (30.8)	516 (35.5)	465 (30.5)	0.002
Chronic liver disease	347 (10.3)	118 (8.1)	189 (12.4)	0.001
Chronic neurological disease	349 (10.4)	244 (16.8)	180 (11.8)	< 0.001
Cardiovascular disease	480 (14.3)	258 (17.8)	267 (17.5)	0.001
Chronic lung disease	320 (9.5)	160 (11.0)	181 (11.9)	0.035
Chronic kidney disease	324 (9.7)	148 (10.2)	154 (10.1)	0.812
Solid malignant tumor	1350 (40.2)	626 (43.1)	663 (43.4)	0.050
Hematologic malignancies	236 (7.0)	113 (7.8)	212 (13.9)	< 0.001
Immunocompromised	134 (4.0)	66 (4.5)	61 (4.0)	0.652
Connective tissue disease	100 (3.0)	32 (2.2)	42 (2.8)	0.317
Type of infection				< 0.001
Community-acquired	2586 (77.1)	1058 (72.8)	1026 (67.2)	
Nursing home-acquired	13 (0.4)	15 (1.0)	7 (0.5)	
Nursing hospital-acquired	60 (1.8)	29 (2.0)	41 (2.7)	
Hospital-acquired	697 (20.8)	351 (24.2)	453 (29.7)	
Site of infection				
Pulmonary	806 (24.0)	536 (36.9)	663 (43.4)	< 0.001
Abdominal	1479 (44.1)	544 (37.4)	502 (32.9)	< 0.001
Urinary tract	638 (19.0)	220 (15.1)	120 (7.9)	< 0.001
Skin and soft tissue	130 (3.9)	65 (4.5)	59 (3.9)	0.589
Catheter-related	27 (0.8)	17 (1.2)	14 (0.9)	0.474
Neurologic	23 (0.7)	15 (1.0)	13 (0.9)	0.453
Treatment and microbiology				
Corticosteroid use	343 (10.2)	261 (18.0)	355 (23.2)	< 0.001
Multidrug-resistant bacteria	564 (16.8)	262 (18.0)	304 (19.9)	< 0.001
Appropriate initial antibiotics	3076 (91.7)	1321 (90.9)	1352 (88.5)	< 0.001
Hospital course				
ICU admission	1150 (34.3)	634 (43.6)	835 (54.7)	< 0.001

Baseline characteristics stratified by in-hospital outcomes (remained non-frail, new-onset frailty, and in-hospital death). Continuous variables are presented as mean (standard deviation) or median (interquartile range), and categorical variables as number (percentage)

Baseline CFS values are presented to reflect premorbid functional status within the non-frail range (CFS < 5)

The distribution of infection characteristics also differed across groups. Pulmonary infection was more common among patients with new-onset frailty and those who died during hospitalization (24.0%, 36.9%, and 43.4%; *p* < 0.001), whereas abdominal infection (44.1%, 37.4%, and 32.9%; *p* < 0.001) and urinary tract infection (19.0%, 15.1%, and 7.9%; *p* < 0.001) were more frequent among patients who remained non-frail. Hospital-acquired infection was more frequent among patients with new-onset frailty and those who died during hospitalization (20.8%, 24.2%, and 29.7%; overall *p* < 0.001).

### Clinical factors associated with new-onset frailty and in-hospital death

**Fig. 3:**
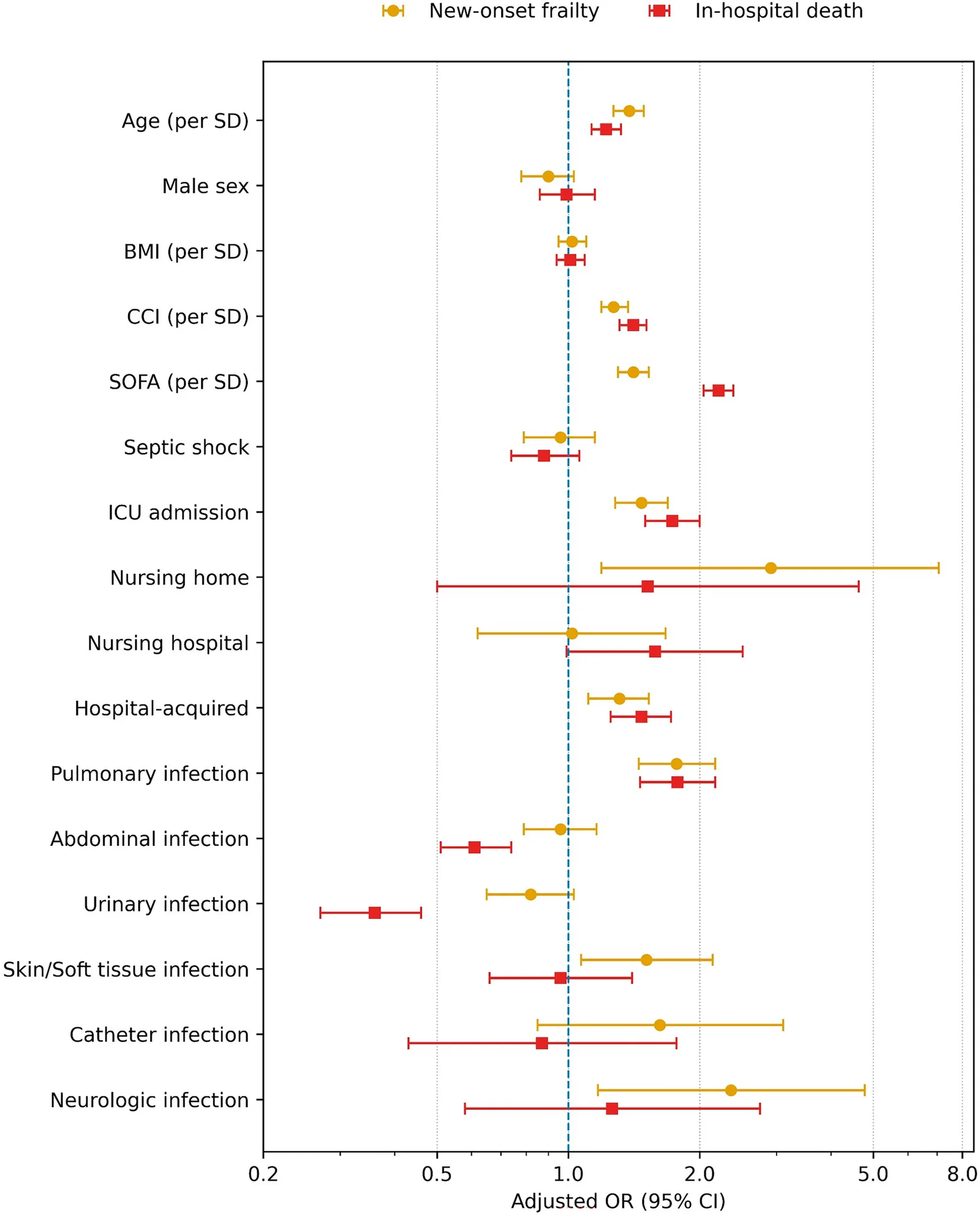
Clinical factors associated with new-onset frailty and in-hospital death. Multinomial logistic regression analysis showing adjusted odds ratios (ORs) and 95% confidence intervals (CIs) for new-onset frailty and in-hospital death, with remaining non-frail at discharge as the reference outcome. Age, body mass index, Charlson Comorbidity Index, and SOFA score were modeled per 1-SD increase. The model was adjusted for prespecified baseline and early clinical covariates. Because the analysis was restricted to patients without baseline frailty, baseline CFS was not included as an adjustment covariate. Estimates are interpreted as adjusted associations rather than causal effects

In multinomial logistic regression analyses with remaining non-frail as the reference outcome and in-hospital death modeled as a competing outcome, several prespecified clinical factors were associated after adjustment with new-onset frailty at discharge (Fig. 3; Supplementary Table 2). Older age was associated with higher odds of new-onset frailty (per 1-SD increase, adjusted OR [aOR], 1.38; 95% CI, 1.27–1.49; *p* < 0.001), as were higher CCI (per 1-SD increase, aOR, 1.27; 95% CI, 1.19–1.37; *p* < 0.001), higher SOFA score (per 1-SD increase, aOR, 1.41; 95% CI, 1.30–1.53; *p* < 0.001), and ICU admission (aOR, 1.47; 95% CI, 1.28–1.69; *p* < 0.001).

Hospital-acquired infection was associated with higher odds of new-onset frailty compared with community-acquired infection (aOR, 1.31; 95% CI, 1.11–1.53; *p* < 0.001). Nursing home-acquired infection was also associated with new-onset frailty (aOR, 2.91; 95% CI, 1.19–7.06; *p* = 0.023), whereas nursing hospital-acquired infection was not (aOR, 1.02; 95% CI, 0.62–1.67; *p* = 0.900). Among infection sites, pulmonary infection (aOR, 1.77; 95% CI, 1.45–2.17; *p* < 0.001), skin/soft tissue infection (aOR, 1.51; 95% CI, 1.07–2.14; *p* = 0.023), and neurologic infection (aOR, 2.36; 95% CI, 1.17–4.78; *p* = 0.014) were associated with higher odds of new-onset frailty. Male sex, BMI, septic shock, abdominal infection, urinary tract infection, and catheter-related infection were not statistically associated with new-onset frailty after adjustment.

The corresponding multinomial estimates for in-hospital death are shown in Fig. 3. The overall pattern suggested that several factors associated with new-onset frailty were also associated with in-hospital death, but the magnitude differed between outcomes. In particular, SOFA score (per SD) showed a larger adjusted association with in-hospital death (aOR, 2.21; 95% CI, 2.04–2.39) than with new-onset frailty (aOR, 1.41; 95% CI, 1.30–1.53).

### Discharge destination among hospital survivors

**Fig. 4:**
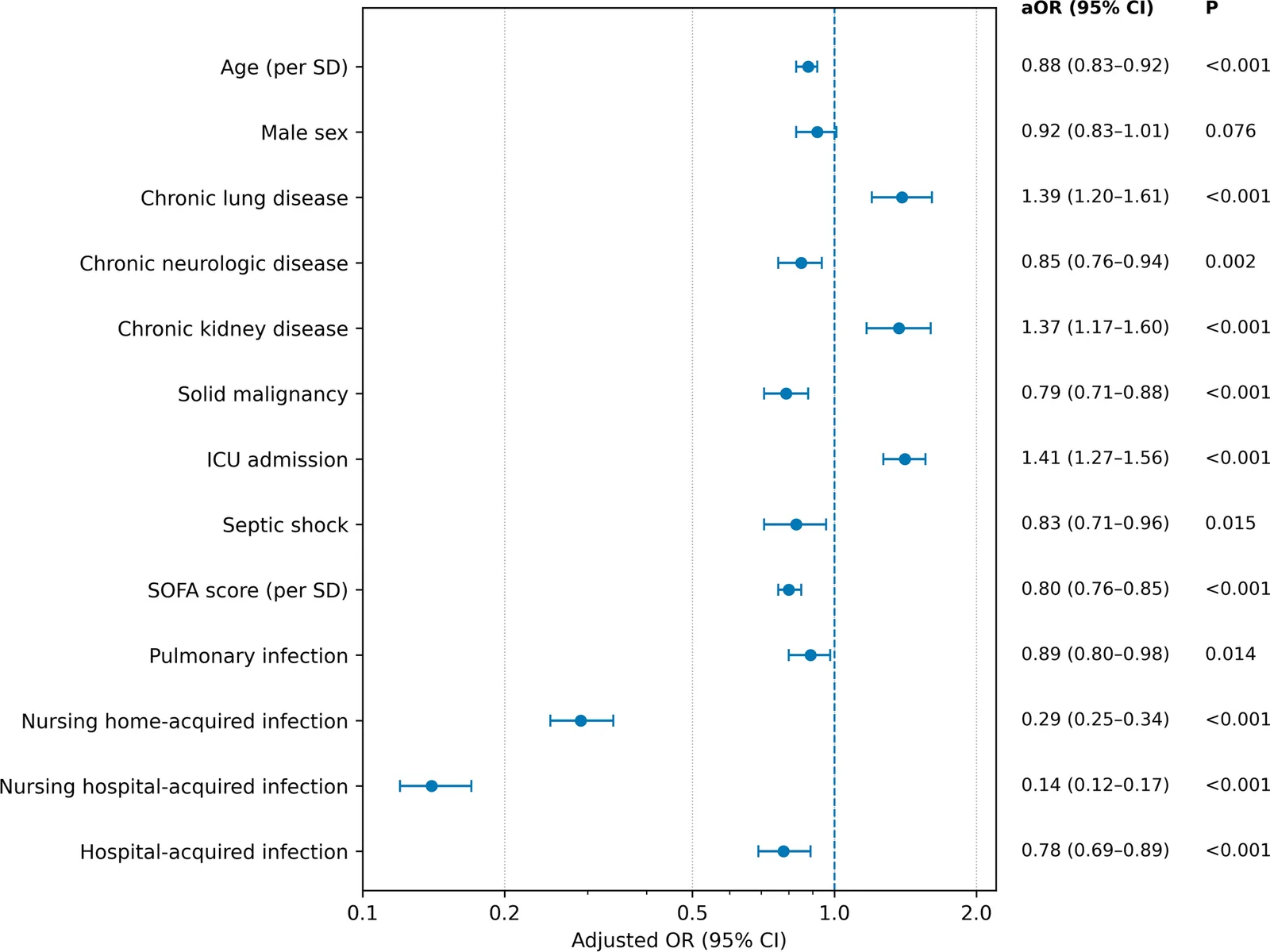
Exploratory analysis of factors associated with home discharge among patients with new-onset frailty. Forest plot showing adjusted odds ratios and 95% confidence intervals for home discharge among hospital survivors with new-onset frailty at discharge. Age and SOFA score were modeled per 1-SD increase; other variables were modeled as binary or categorical variables. This exploratory analysis was restricted to patients with new-onset frailty

Among hospital survivors, 42.8% of patients with new-onset frailty were discharged home, compared with 86.4% of those who remained non-frail at discharge (Fig. 2B). In exploratory analyses restricted to patients with new-onset frailty, clinical factors associated with home discharge are shown in Fig. 4. Older age, chronic neurologic disease, solid malignancy, septic shock, higher SOFA score, pulmonary infection, and nursing home-, nursing hospital-, or hospital-acquired infection were associated with lower odds of home discharge, whereas chronic lung disease, chronic kidney disease, and ICU admission were associated with higher odds of home discharge.

### Sensitivity analyses

In sensitivity analyses using IPCW to account for potential selection by survival to discharge, the direction and magnitude of associations between key clinical factors and new-onset frailty were generally consistent with the primary analysis (Supplementary Table 3). In the IPCW-weighted model, ICU admission (OR, 1.37; 95% CI, 1.18–1.59), hospital-acquired infection (OR, 1.27; 95% CI, 1.06–1.50), pulmonary infection (OR, 1.78; 95% CI, 1.43–2.22), skin/soft tissue infection (OR, 1.65; 95% CI, 1.15–2.36), neurologic infection (OR, 2.53; 95% CI, 1.23–5.20), and nursing home-acquired infection (OR, 3.03; 95% CI, 1.12–8.17) remained associated with new-onset frailty.

In exploratory subgroup analyses stratified by ICU admission and septic shock status, the overall direction of key associations with new-onset frailty was broadly similar to that observed in the primary analysis (Supplementary Table 4). Older age, higher SOFA score, and pulmonary infection were consistently associated with higher odds of new-onset frailty across ICU and non-ICU patients and across patients with and without septic shock. Some estimates were less precise in smaller strata, particularly for less frequent infection acquisition settings and infection sites.

## Discussion

In this multicenter cohort study of patients hospitalized with sepsis, we evaluated new-onset frailty among patients who were non-frail at baseline. Three main findings emerged. First, new-onset frailty at discharge was common, occurring in 22.9% of patients, while 24.1% died during hospitalization. Second, in multinomial models that included in-hospital death as a competing outcome, older age, greater comorbidity burden, higher illness severity, ICU admission, and pulmonary infection were associated after adjustment with higher odds of new-onset frailty. These factors were also associated with in-hospital death, although illness severity showed a stronger association with death. Third, among hospital survivors, patients with new-onset frailty were less likely to be discharged home than those who remained non-frail, although 42.8% of patients with new-onset frailty were discharged home.

Our findings should be interpreted in the context of prior work on frailty transitions and functional recovery after critical illness. Gill et al. established frailty as a dynamic condition in community-living older adults, showing frequent transitions between frailty states over time [9]. In critical illness, Ferrante et al. described functional trajectories before and after ICU admission, while Brummel et al. showed that frailty after critical illness is often newly acquired and may change during the first year after hospitalization [10, 11]. Fuest et al. further evaluated intrahospital functional trajectories in frail and non-frail critically ill patients, and the FORECAST study recently assessed frailty trajectories and care processes during recovery from critical illness using the CFS and Frailty Index [12, 13]. Our study extends this literature in several ways. First, we focused specifically on patients hospitalized with sepsis who were non-frail before the onset of sepsis. Second, we evaluated frailty newly observed at hospital discharge, thereby capturing functional vulnerability that emerged during the acute hospitalization phase rather than after post-discharge recovery or rehabilitation. Third, because discharge frailty can be observed only among survivors, we incorporated in-hospital death as a mutually exclusive competing outcome rather than restricting the primary analysis to hospital survivors alone.

A key methodological feature of our analysis was the inclusion of in-hospital death as a mutually exclusive outcome. Because discharge frailty status can be observed only among hospital survivors, excluding patients who died may provide an incomplete view of outcomes after sepsis. By using a multinomial framework, we evaluated new-onset frailty and in-hospital death within the same analytic cohort. This approach showed that both functional decline and mortality were frequent among patients who were non-frail before sepsis, supporting the importance of interpreting discharge frailty in the broader context of in-hospital outcomes [15–17].

Several clinical factors were associated with new-onset frailty. Older age and greater comorbidity burden may reflect reduced physiologic reserve, whereas higher SOFA score, pulmonary infection, and ICU admission may reflect the burden of acute illness and intensive treatment [18–20]. The overlap between factors associated with new-onset frailty and those associated with in-hospital death suggests that these outcomes share common clinical features, although they may represent different consequences of sepsis hospitalization. These findings suggest that new-onset frailty at discharge reflects both pre-existing vulnerability within the non-frail range and the impact of acute illness during hospitalization. The exploratory subgroup analyses stratified by ICU admission and septic shock status suggested that the main associations, particularly those for older age, SOFA score, and pulmonary infection, were broadly consistent across clinically relevant acute-severity subgroups. However, because some subgroup estimates were imprecise, these findings should be interpreted as supportive rather than definitive.

The association between new-onset frailty at discharge and home discharge should be interpreted with caution. Discharge frailty status and discharge destination are both determined near the end of hospitalization and may be influenced by overlapping clinical and non-clinical factors, including illness severity, the hospital course, rehabilitation exposure, discharge planning, family support, availability of post-acute care, and healthcare system practices. Therefore, we do not interpret discharge frailty as a causal determinant of home discharge. Rather, the discharge destination analysis was intended to evaluate the clinical relevance of new-onset frailty among hospital survivors and was interpreted as a survivor-conditional association.

The interpretation of home discharge also requires consideration of the Korean healthcare context. In South Korea, nursing hospitals often function as post-acute care facilities and may provide ongoing rehabilitation, caregiver support, or medical care after acute hospitalization. Therefore, classification of discharge to a nursing hospital as non-home discharge should not be interpreted as equivalent to poor functional recovery in all cases. Conversely, home discharge may also be influenced by family caregiving capacity and social support, not solely by functional status. These healthcare system factors may differ from discharge practices in Western healthcare systems and should be considered when interpreting the association between new-onset frailty and discharge destination.

From a clinical perspective, assessment of frailty at both admission and discharge may help characterize functional decline during hospitalization and identify patients who may require individualized recovery planning. However, our findings should not be interpreted as evidence that routine frailty assessment alone improves outcomes or that discharge frailty can be used as a standalone decision-making tool. Instead, discharge frailty may serve as one component of a broader clinical assessment that includes illness severity, baseline health status, rehabilitation needs, caregiver support, and post-acute care resources. Future studies with longitudinal post-discharge frailty measurements and detailed rehabilitation data are needed to determine whether patients with new-onset frailty represent a target population for structured rehabilitation or transitional-care interventions [21–25].

Several limitations should be acknowledged. First, this was an observational study, and the reported estimates should be interpreted as adjusted associations rather than causal effects. Residual confounding from unmeasured factors, including baseline physical performance, socioeconomic status, family support, rehabilitation intensity, and hospital-level discharge practices, may remain. In particular, the absence of detailed data on in-hospital rehabilitation, physical therapy exposure, timing, and intensity is a major limitation, because differences in rehabilitation practices across patients and centers may have contributed to the observed development of frailty. Second, discharge frailty status was observable only among hospital survivors. Although in-hospital death was incorporated as a competing outcome and IPCW sensitivity analyses were performed, residual selection related to survival cannot be fully excluded. Third, frailty assessment using the CFS may be subject to measurement variability. The KSA registry used standardized 1-to-9 CFS instructions and registry data collection training that included CFS coding procedures; however, formal CFS-specific certification, internal validation, and inter-rater reliability testing across centers were not performed. Baseline CFS was also based on retrospective assessment of premorbid function using patient or caregiver interview and medical record review, which may have introduced recall error or misclassification. Fourth, longitudinal frailty measurements after discharge were not available, and we could not determine whether new-onset frailty represented transient impairment or persistent frailty. Fifth, this study was conducted within a Korean multicenter registry composed mainly of tertiary or university-affiliated hospitals. Therefore, the findings may not be directly generalizable to community hospitals, lower-resource settings, or healthcare systems with different rehabilitation, discharge planning, and post-acute care structures.

Despite these limitations, this study used a large, multicenter sepsis registry with standardized data collection, allowing evaluation of new-onset frailty in a broad cohort of patients hospitalized with sepsis. By focusing on patients without baseline frailty and incorporating in-hospital death as a competing outcome, we characterized frailty newly observed at discharge among patients who were non-frail before sepsis.

## Conclusions

New-onset frailty and in-hospital death were common among patients hospitalized with sepsis who were non-frail at baseline. New-onset frailty was associated with reduced likelihood of home discharge among survivors. Assessment of frailty at admission and discharge may help characterize functional decline and inform discharge planning after sepsis.

## Supplementary Information

Additional file1 (DOCX 32 KB)

## Data Availability

The data that support the findings of this study were obtained from the Korean Sepsis Alliance (KSA) registry. The data are not publicly available due to institutional and ethical restrictions but are available from the corresponding author upon reasonable request and with permission of the Korean Sepsis Alliance and relevant institutional review boards.

## Abbreviations

BMI: Body mass index
CCI: Charlson Comorbidity Index
CFS: Clinical Frailty Scale
CI: Confidence interval
ICU: Intensive care unit
IPCW: Inverse probability of censoring weighting
KSA: Korean sepsis alliance
OR: Odds ratio
SOFA: Sequential organ failure assessment
