# Supplementary material for: In-hospital development of new-onset frailty and discharge outcomes among patients with sepsis: a Korean multicenter registry study
Source: J Intensive Care. 2026 Jul 13;14:78. doi: 10.1186/s40560-026-00907-1 (PMC13504832; doi:10.1186/s40560-026-00907-1)
Supplement: Supplementary file 1 [file 40560_2026_907_MOESM1_ESM.docx]

**Supplementary Material**

In-Hospital Development of New-Onset Frailty and Discharge Outcomes Among Patients With Sepsis: A Korean Multicenter Registry Study

Authors

Eunjeong Choi, MD^1^; Kipoong Kim, PhD^2^; Woo Hyun Cho, MD^1,3^; Jin Ho Jang, MD^1,3^; Ryoung-Eun Ko, MD^4^; Gee Young Suh, MD^4^; Chae Man Lim, MD^5^; Hye Ju Yeo, MD, PhD^1,3^; On behalf of the Korean Sepsis Alliance (KSA) investigators

**Supplementary Table 1. Institutional review board approval numbers for participating institutions**

| Institution | Institutional review boards number | Approval date | Study title |
| --- | --- | --- | --- |
| Asan Medical Center | 2018-0678 | 2018.6.11 | Korean Sepsis Alliance registry: A multicenter observational cohort study |
| Chonnam national university hospital | CNUH-2019-075 | 2019.3.13 | Korean Sepsis Alliance registry: A multicenter observational cohort study |
| Chungnam national university hospital | 2019-11-048-021 | 2019.12.19 | Korean Sepsis Alliance registry: A multicenter observational cohort study |
| Chungnam National University Sejong Hospital | 2020-08-017 | 2020.8.27 | Korean Sepsis Alliance registry: A multicenter observational cohort study |
| Hallym University Sacred heart hospital | 2018-09-004 | 2018.10.16 | Korean Sepsis Alliance registry: A multicenter observational cohort study |
| Hanyang University Guri Hospital | 2020-08-015 | 2020.8.27 | Korean Sepsis Alliance registry: A multicenter observational cohort study |
| Inje University ilsan Paik Hospital | 2021-08-003 | 2021.09.07 | Korean Sepsis Alliance registry: A multicenter observational cohort study |
| Inje University Sanggye Paik Hospital | 2018-08-014 | 2018.9.12 | Korean Sepsis Alliance registry: A multicenter observational cohort study |
| Jeju National University Hospital | 2018-06-012 | 2018.7.17 | Korean Sepsis Alliance registry: A multicenter observational cohort study |
| Jeonbuk national university hospital | CUH 2018-10-027 | 2019.3.13 | Korean Sepsis Alliance registry: A multicenter observational cohort study |
| Kangwon national university hospital | 2018-08-004-001 | 2018.11.7 | Korean Sepsis Alliance registry: A multicenter observational cohort study |
| Pusan National University Yangsan Hospital | 05-2019-092 | 2019.7.11 | Korean Sepsis Alliance registry: A multicenter observational cohort study |
| Samsung Medical Center | 2024-06-010 | 2024.06.14 | Korean Sepsis Alliance registry: A multicenter observational cohort study |
| Seoul National University Bundang Hospital | B-181-500-402 | 2018.10.11 | Korean Sepsis Alliance registry: A multicenter observational cohort study |
| Seoul National University Hospital | 1808-135-967 | 2018.9.19 | Korean Sepsis Alliance registry: A multicenter observational cohort study |

**Supplementary Table 2. Multinomial logistic regression analysis for new-onset frailty among patients without baseline frailty**

| **Variable** | **New-onset Frailty adjusted OR (95% CI)** | **In-hospital Death adjusted OR (95% CI)** |
| --- | --- | --- |
| Age (per SD) | 1.38 (1.27–1.49) | 1.22 (1.13–1.32) |
| Male sex | 0.90 (0.78–1.03) | 0.99 (0.86–1.15) |
| BMI (per SD) | 1.02 (0.95–1.10) | 1.01 (0.94–1.09) |
| CCI (per SD) | 1.27 (1.19–1.37) | 1.41 (1.31–1.51) |
| SOFA (per SD) | 1.41 (1.30–1.53) | 2.21 (2.04–2.39) |
| Septic shock | 0.96 (0.79–1.15) | 0.88 (0.74–1.06) |
| ICU admission | 1.47 (1.28–1.69) | 1.73 (1.50–2.00) |
| Community (reference) | *Reference* | *Reference* |
| Nursing home | 2.91 (1.19–7.06) | 1.52 (0.50–4.63) |
| Nursing hospital | 1.02 (0.62–1.67) | 1.58 (0.99–2.51) |
| Hospital-acquired | 1.31 (1.11–1.53) | 1.47 (1.25–1.72) |
| Pulmonary infection | 1.77 (1.45–2.17) | 1.78 (1.46–2.17) |
| Abdominal infection | 0.96 (0.79–1.16) | 0.61 (0.51–0.74) |
| Urinary infection | 0.82 (0.65–1.03) | 0.36 (0.27–0.46) |
| Skin/Soft tissue infection | 1.51 (1.07–2.14) | 0.96 (0.66–1.40) |
| Catheter infection | 1.62 (0.85–3.11) | 0.87 (0.43–1.77) |
| Neurologic infection | 2.36 (1.17–4.78) | 1.26 (0.58–2.75) |

Adjusted ORs and 95% CIs for new-onset frailty and in-hospital death were derived from a multinomial logistic regression model, with remaining non-frail at discharge as the reference outcome. The model included prespecified clinical covariates. Age, BMI, CCI, and SOFA score were modeled per 1-SD increase.

**Supplementary table 3. IPCW sensitivity analysis for new-onset frailty among hospital survivors**

| **Variable** | **Primary analysis adjusted OR (95% CI)** | **IPCW-adjusted OR (95% CI)** |
| --- | --- | --- |
| Age (per SD) | 1.38 (1.27–1.49) | 1.44 (1.32–1.57) |
| Male sex | 0.90 (0.78–1.03) | 0.86 (0.75–1.00) |
| BMI (per SD) | 1.02 (0.95–1.10) | 1.03 (0.95–1.11) |
| CCI (per SD) | 1.27 (1.19–1.37) | 1.18 (1.09–1.27) |
| SOFA (per SD) | 1.41 (1.30–1.53) | 1.38 (1.26–1.51) |
| Septic shock | 0.96 (0.79–1.15) | 0.98 (0.80–1.20) |
| ICU admission | 1.47 (1.28–1.69) | 1.37 (1.18–1.59) |
| Community (reference) | *Reference* | *Reference* |
| Nursing home | 2.91 (1.19–7.06) | 3.03 (1.12–8.17) |
| Nursing hospital | 1.02 (0.62–1.67) | 0.93 (0.53–1.63) |
| Hospital-acquired | 1.31 (1.11–1.53) | 1.27 (1.06–1.50) |
| Pulmonary infection | 1.77 (1.45–2.17) | 1.78 (1.43–2.22) |
| Abdominal infection | 0.96 (0.79–1.16) | 0.96 (0.77–1.18) |
| Urinary infection | 0.82 (0.65–1.03) | 0.81 (0.64–1.04) |
| Skin/Soft tissue infection | 1.51 (1.07–2.14) | 1.65 (1.15–2.36) |
| Catheter infection | 1.62 (0.85–3.11) | 1.62 (0.77–3.40) |
| Neurologic infection | 2.36 (1.17–4.78) | 2.53 (1.23–5.20) |

Sensitivity analysis using inverse probability of censoring weighting (IPCW) to account for survival to discharge. Adjusted ORs and 95% CIs are presented for the primary model and IPCW-adjusted model. The outcome was new-onset frailty compared with remaining non-frail. Age, BMI, CCI, and SOFA score were modeled per 1-SD increase. Community-acquired infection was used as the reference category for infection acquisition setting.

**Supplementary Table 4. Exploratory subgroup analyses for new-onset frailty stratified by ICU admission and septic shock status**

| **Variable** | **Non-ICU aOR (95% CI)** | **ICU aOR (95% CI)** | **No septic shock aOR (95% CI)** | **Septic shock aOR (95% CI)** |
| --- | --- | --- | --- | --- |
| Age (per SD) | 1.33 (1.20-1.48) | 1.49 (1.32-1.68) | 1.35 (1.24-1.48) | 1.54 (1.28-1.85) |
| Male sex | 0.82 (0.68-0.98) | 1.01 (0.82-1.26) | 0.90 (0.77-1.05) | 0.89 (0.64-1.22) |
| BMI (per SD) | 1.04 (0.93-1.16) | 0.32 (0.11-0.93) | 1.03 (0.94-1.14) | 0.67 (0.18-2.47) |
| CCI (per SD) | 1.40 (1.27-1.54) | 1.11 (0.98-1.26) | 1.38 (1.27-1.50) | 0.97 (0.82-1.16) |
| SOFA score (per SD) | 1.48 (1.32-1.66) | 1.36 (1.21-1.53) | 1.41 (1.28-1.55) | 1.42 (1.22-1.66) |
| Septic shock | 0.93 (0.71-1.24) | 0.97 (0.76-1.25) | — | — |
| ICU admission | — | — | 1.52 (1.29-1.78) | 1.30 (0.95-1.79) |
| Community (reference) | *Reference* | *Reference* | *Reference* | *Reference* |
| Nursing home | 2.50 (0.78-7.97) | 3.26 (0.77-13.84) | 2.59 (0.92-7.30) | 3.89 (0.66-22.90) |
| Nursing hospital | 1.01 (0.53-1.90) | 1.02 (0.46-2.28) | 1.08 (0.61-1.91) | 0.85 (0.32-2.27) |
| Hospital-acquired | 1.20 (0.95-1.51) | 1.42 (1.13-1.78) | 1.30 (1.08-1.57) | 1.44 (1.04-2.00) |
| Pulmonary infection | 1.62 (1.24-2.13) | 1.93 (1.43-2.62) | 1.68 (1.34-2.10) | 2.17 (1.37-3.43) |
| Abdominal infection | 0.95 (0.74-1.23) | 0.97 (0.72-1.32) | 0.87 (0.70-1.09) | 1.32 (0.88-1.97) |
| Urinary tract infection | 0.89 (0.66-1.20) | 0.75 (0.53-1.07) | 0.74 (0.58-0.96) | 1.19 (0.72-1.98) |
| Skin/soft tissue infection | 1.37 (0.85-2.19) | 1.79 (1.05-3.04) | 1.35 (0.91-1.99) | 2.55 (1.14-5.69) |
| Catheter infection | 2.52 (1.08-5.85) | 0.98 (0.35-2.75) | 1.52 (0.69-3.34) | 2.07 (0.65-6.61) |
| Neurologic infection | 3.62 (1.28-10.28) | 1.75 (0.68-4.56) | 2.67 (1.27-5.64) | 0.65 (0.06-6.59) |

Values are adjusted odds ratios and 95% confidence intervals for new-onset frailty compared with remaining non-frail at discharge. Estimates were derived from multinomial logistic regression models, with remaining non-frail as the reference outcome and in-hospital death modeled as a competing outcome. Models included the same prespecified covariates as the primary analysis, except that the corresponding stratifying variable was omitted from each subgroup model. Age, BMI, CCI, and SOFA score were modeled per 1-SD increase. Community-acquired infection was used as the reference category for infection acquisition setting. These subgroup analyses were exploratory and were interpreted qualitatively because some strata contained smaller numbers of events.
